# Student learning outcomes from a pilot medical innovations course with nursing, engineering, and biology undergraduate students

**DOI:** 10.1186/s40594-017-0095-y

**Published:** 2017-11-27

**Authors:** Patrice M. Ludwig, Jacquelyn K. Nagel, Erica J. Lewis

**Affiliations:** 1 Department of Biology, James Madison University, 951 Carrier Dr. MSC 7801, Harrisonburg, VA 22807 USA; 2 Department of Engineering, James Madison University, 801 Carrier Drive MSC 4113, Harrisonburg, VA 22807 USA; 3 School of Nursing, James Madison University, 820 Madison Drive MSC 4305, Harrisonburg, VA 22807 USA

**Keywords:** Innovative education, Multidisciplinary education, MakerSpace, STEM, Technology, Healthcare, 3D printing, Qualitative content analysis, Creativity

## Abstract

**Background:**

Preparing today’s undergraduate students from science, technology, engineering, and math (STEM) and related health professions to solve wide-sweeping healthcare challenges is critical. Moreover, it is imperative that educators help students develop the capabilities needed to meet those challenges, including problem solving, collaboration, and an ability to work with rapidly evolving technologies. We piloted a multidisciplinary education (ME) course aimed at filling this gap, and subsequently assessed whether or not students identified achieving the course objectives. In the course, undergraduate students from engineering, pre-nursing (students not yet admitted to the nursing program), and pre-professional health (e.g., pre-med and pre-physician’s assistant) were grouped based on their diversity of background, major, and StrengthsFinder® proficiencies in a MakerSpace to create tangible solutions to health-related problems facing the community. We then used qualitative content analysis to assess the research question: what is the impact of undergraduate multidisciplinary education offered in a MakerSpace on student attitudes towards and perceptions of skills required in their own as well as others occupations?

**Results:**

We discovered these students were able to identify and learn capabilities that will be critical in their future work. For example, students appreciated the challenging problems they encountered and the ability to meet demands using cutting-edge technologies including 3D printers. Moreover, they learned the value of working in a multidisciplinary group. We expected some of these findings, such as an increased ability to work in teams. However, some themes were unexpected, including students explicitly appreciating the method of teaching that focused on experiential student learning through faculty mentoring.

**Conclusions:**

These findings can be used to guide additional research. Moreover, offering a variety of these courses is a necessary step to prepare students for the current and future workforce. Finally, these classes should include a focus on intentional team creation with the goal of allowing students to solve challenging real-world problems through ethical reasoning and collaboration.

**Electronic supplementary material:**

The online version of this article (10.1186/s40594-017-0095-y) contains supplementary material, which is available to authorized users.

## Background

Many of today’s STEM (science, technology, engineering, and math) students will be tomorrow’s professionals responsible for solving an increasingly wide number of health problems. Current issues, such as global emerging infectious diseases and the opioid crisis, are both critical and convoluted enough such that no discipline or profession will be able to solve them alone; diverse teams working together will address these burdens. It is in the context of this diversity and collaboration that innovation is most likely to occur (Doorley and Witthoft 2011). While much of this work is happening at the professional level, current undergraduate students will eventually join the workforce. Thus, we must create educational experiences for students to learn the skills to work collaboratively across boundaries in order to appropriately train future problem solvers.

A critical concern regarding creating solutions to these perplexing problems is that the available technology seems to advance as quickly as the problems themselves. Many current healthcare professionals lack training to work with emerging technologies, thereby stalling advances in public health and industry (Powell-Cope et al. 2008; Miller et al. 2000; Crebert et al. 2004; Vilmante and Ramune 2008).

Additionally, although employers identify problem-solving skills as top priority when hiring (American Management Association 2012), they also report that many job applicants today lack training in the process of problem solving or even identifying problems (Lowden et al. 2011). Thus, there is a need to train STEM and related health profession undergraduate students to integrate novel technologies and to solve problems. It is imperative to adapt their education to include opportunities that will enable these students to develop the capabilities, including the knowledge, skills, attitudes, and behaviors needed for working in environments with multiple professionals collaborating on issues.

Opportunities exist for professionals to develop problem solving, collaboration, and innovation skills (Van Note 2006; Cotner et al. 2013; Muthyala and Wei 2012; Ellis and Goodyear 2016). However, there are many fewer opportunities for undergraduate students to cultivate these proficiencies before they are deeply embedded in their chosen profession (McClelland and Kleinke 2013; Spoelstra et al. 2014). This lack of opportunity for undergraduate students to engage in these activities, on our campus and at other institutions of higher education, provided a motivation for our work. We posit that initiating strategic multidisciplinary education (ME) (described below) early in the post-secondary educational timeline will allow undergraduate students from STEM and related health professions to develop capabilities that they can later build upon as they enter professional training, graduate training, and the workforce. Educating pre-professional students in processes of creativity and innovation is recognized and encouraged to enhance innovation in addressing current health challenges (Ness 2011). We piloted an innovative course that allowed undergraduate students from engineering, students who will enter the nursing program in their junior year (hereafter “pre-nursing”), and students from biology and health-sciences majors with pre-professional designations (e.g., pre-medical, pre-physician’s assistant, or pre-dental) to work in diverse teams.

Teams in the course focused on identifying problems and developing solutions to a local community health challenge in a technological environment, a MakerSpace. This is a physical place where people gather to create and learn (Whitmer 2016) in which manufacturing equipment such as 3D printers, laser cutters, milling machines, and hand tools are available for the process of designing and prototyping (Levy et al. 2016). This creative setting provides undergraduate students with technology to foster problem solving, collaboration, and proficiency of newly available technology.

We designated our course as multidisciplinary education (ME). We reviewed literature to assess terms related to our student teams and their work in our course. Terms evaluated included interprofessional, multidisciplinary, interdisciplinary, trans-disciplinary, and transdisciplinary. We concluded that there is currently no term with a definition that explicitly fits the mix of students and type of experiences in our course. While this is evidence to the innovative nature of the course, it creates a problem that warrants some attention. The terms that most closely aligned with our work were interprofessional education (IPE) and multidisciplinary education (ME). Training students from different pre-professions to work collaboratively on issues is specifically defined in the healthcare industry. The World Health Organization defines IPE occurring when students from at least two professions learn “about, from, and with each other” towards the result of improving health outcomes for patients, families, or communities (p. 7). This definition is not without problems related to our work. First, there is a lack of clarity in this definition as to when in the educational trajectory a student can be considered “from” a profession. Is it when they begin their professional training, when they are accepted into a major as an undergraduate, or when they have declared a major? Further, this definition is limited by only including professions. What about disciplines? The term multidisciplinary, as defined by the Oxford Dictionary, does include professions and disciplines: “Combining or involving several academic disciplines or professional specializations in an approach to a topic or problem” but it lacks the component of learning “about, from, and with each other” that is foundational to our course formation and the course focus on patient outcomes. Multidisciplinary education, however, better aligns with our course. The term focuses on developing a deeper understanding of one’s own area of study and learning to communicate across disciplines; it focuses less on actually integrating professional practices (Pirrie et al. 1999). Thus, we use the term multidisciplinary education (ME) to best reflect our mix of students and educational experiences. Although we use ME to define the course work and subsequent research, the term “interprofessional” is retained when necessary to accurately reflect literature cited.

### Motivation for the course and the research

The motivation for designing and teaching the course came from the instructors and the MakerSpace director observing an opportunity. Teaching the course in a MakerSpace offers an environment for working on open-ended problems that affect our local community. The desire to focus on the local community came from our personal alignment with our university’s focus on community engagement. The instructors worked collaboratively before the course to identify what successful students would know and be able to do upon exiting the course, according to backward design principles (Wiggins and McTighe 2005). The objectives of professional organizations such as the Interprofessional Education Collaborative (IPEC) align with and are mapped to our course objectives (Table 1). The IPEC was established in 2011 by a collaborative expert panel (IPE Collaborative Expert Panel) and updated in 2016 to describe core competencies for interprofessional collaborative practice for the healthcare disciplines (IPE Collaborative 2016). The domains include the overarching interprofessional collaboration, values/ethics, role/responsibilities, interprofessional communication practices, and teamwork/team-based practice to promote unified taxonomy, workspaces, technology, and educational activities and evaluation (2016). IPEC continues to grow in both esteemed membership and impact, including the American Psychological Association (APA), American Council of Academic Physical Therapy (ACAPT), Association of American Veterinary Medical Colleges (AAVMC), and the Council on Social Work Education (CSWE). Thus, the alignment is appropriate given that the goal of the students in the course is to enter healthcare or engineering as professionals.

**Table 1:** Table of course level objectives and the corresponding Interprofessional Education Collaborative (IPEC) competency they align with in parentheses

Apply problem solving skills to an actual community health challenge for vulnerable populations. (Values/ethics, roles/responsibilities)
Use collaboration skills to work together with a group of diverse pre-professionals. (Interprofessional communication, teams and teamwork)
Engage in self-analysis to identify systemic factors relevant to supporting quality teamwork. (Roles/responsibilities, teams and teamwork)
Evaluate personal strengths and their applications to leadership and participation multidisciplinary teams. (Roles/responsibilities, teams and teamwork)
Evaluate feasibility, potential impact, and limitations of potential solutions. (None)
Discuss the ethical, legal, and practical implications of applying novel technologies, particularly for use with vulnerable populations. (Values/ethics)
Produce and communicate a tangible product using MakerSpace technology that has the potential to improve a community health challenge. (Interprofessional communication, teams and teamwork)

As we searched for an instrument that would allow us to determine the degree to which students achieved the course objectives, it became clear that there was not one that assessed our combination of objectives and mix of students. Thus, a qualitative analysis of an oral exit question was used to determine if students had the perception of achieving the course objectives.

A call has been issued for future research to explore the impact of various student compositions on collaborative learning as well as conduct studies in different contexts and spaces to strengthen the validity of findings (Van den Bossche et al. 2006; Ketcherside et al. 2017). Our study addresses both by using qualitative methods to understand how the intentional recruitment of pre-nursing, engineering, and pre-professional health undergraduate students into a problem-based course situated in a MakerSpace affected students’ achievement of the course objectives. The motivation for the *course* is rooted in exploring collaborative learning in a MakerSpace, the motivation for the *research* is rooted in assessing student perceptions of the multidisciplinary course and their achievement of the course objectives.

### Existing literature

**Fig. 1:**
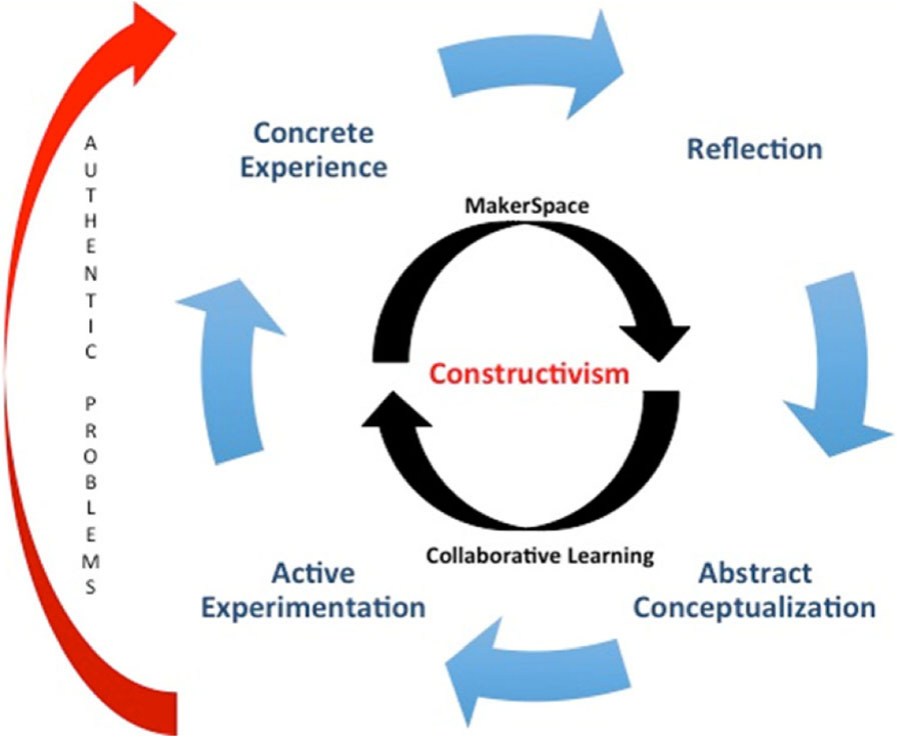
A conceptual model of the relationship between the instructional theory, instructional strategies, and instructional framework for the course that led to the research of assessment of students’ perceptions of achieving the course objectives

The instructional strategies used in the course, collaborative learning, authentic problems, and use of the MakerSpace are all rooted in the constructivist theory and give rise to the instructional framework of the Kolb learning cycle (Fig. 1).

#### Kolb learning cycle

During the course design process, the instructors identified common learning experiences in the representative disciplines as being learning by doing such as laboratory, clinical, and research experiences. The instructors each value the role of reflection in the learning process and aimed to explicitly include this in the course through weekly journaling and through whole class debriefing on class activities. We discovered that the Kolb learning cycle (Kolb 1984) had aspects common to each of the disciplines and incorporated learning through experience as well as the intentional role of reflection. This learning cycle describes experiential learning in four stages: concrete experience, reflective observation, abstract conceptualization, and active experimentation, which flows back to concrete experience. It is rooted in constructivist theory of learning (Kolb 2014) and has been used successfully in undergraduate engineering and undergraduate nursing education. We explicitly mapped our learning activities to the Kolb cycle (Table 2).

**Table 2:** Course activities aligned with the Kolb learning cycle (summarized from Nagel, Lewis, and Ludwig 2017)

Concrete experience	Students select and work in multidisciplinary teams to gain empathy for members of the community with a chronic illness, such as metabolic syndrome. Students interact with and get feedback consistently and frequently with members of their team and members of the patient community throughout the experience on iterations of solutions.
Reflective observation	Students participate in weekly reflective journaling individually and in guided group discussions where they respond to prompts about their process and their developing solutions. There is an oral final where students reflect on the concepts required to maintain a creative design process and produce a quality product in the course.
Abstract conceptualization	Students learn in ways that are different from their typical experience. Engineering students often comment on how much they enjoyed the opportunity to work with people from outside their major and share their knowledge of the design process. Pre-nursing students often comment on their ability to work with technologies and contribute to the design process. Biology students often comment on increasing their creativity and learning processes for problem solving.
Active experimentation	Throughout the course students make prototypes of their product to gain useful feedback from community members. They begin with basic prototypes and iteratively make changes based on feedback from the patient community. They culminate by presenting a beta prototype to stakeholders.

Traditionally, experiential education is considered to be experiences beyond the classroom and curriculum such as internships, study abroad, service learning, and field experiences (Katula and Threnhauser 1999). Kolb’s definition [Learning is the process whereby knowledge is created through the transformation of experience (Kolb 1984, p. 38)], however, does not explicitly state that the experience needs to be outside the classroom. In fact, extending this definition has allowed many disciplines to incorporate reflection into classroom experiences. Abdulwahed and Nagy (2009) showed that students in an undergraduate engineering laboratory experience redesigned for experiential learning had greater learning gains than students in the control laboratory experience. A hallmark of nursing undergraduate education, the clinical experience, is rooted in experiential learning. Lisko and O’Dell (2010) found that experiential learning helped students integrate knowledge during experiences using simulations. Published literature of biology undergraduate education has rarely incorporated the reflective observation component of the Kolb learning cycle (but see Bauerle and Park 2012 and Millenbah and Millspaugh 2003). However, the recent focus on the importance of students learning about their own learning, also known as metacognition (Tanner 2012), suggests that explicitly applying the Kolb learning cycle to biology education is appropriate.

#### Collaborative learning

Collaborative learning is largely rooted in Vygotsky’s sociocultural or social development theory, linking learning within the experiential context and interaction with peers (Vygotsky 1978). Early research suggested that learners educated in a group setting and who cooperate in order to attain common goals are more likely to be successful (May et al. 1937). Since then, research has shown that collaborative learning teams can a range of outcomes from student development (Cabrera et al. 2002) to product development (Edmondson and Nembhard 2009). Collaborative learning situates into the Kolb learning cycle in that our students have a concrete experience together in class, they reflect independently, but then regroup to conceptualize their experience and experiment as a team (Table 1).

Collaborative learning has been shown to improve learning gains in engineering students. A survey of 150 engineering students indicated a significant positive correlation between collaborative learning, self-efficacy, knowledge building, and course grade. Moreover, regardless of reported self-efficacy levels, students who worked with peers earned higher grades than those who reported studying and working independently outside class (Stump et al. 2011). A follow-up study of 513 engineering students revealed females utilized this strategy significantly more than males (Stump et al. 2011). Similarly, a study of 480 engineering students across six schools indicated collaboration with peers produced statistically significant gains relative to traditional instructional methods (Terenzini et al. 2001). Despite this evidence, much of the engineering curriculum is still organized around student retention of facts and skills (Stump et al. 2011).

As most nurses will work on teams with doctors, therapists, and other healthcare professionals to coordinate patient-centered healthcare (Bureau of Labor Statistics 2016), it is imperative that pre-nursing and pre-licensure nursing undergraduate students are taught interprofessional collaboration skills (Ketcherside et al. 2017). Chan and Wood (2012) assert that an early and continual focus on IPE is the best way to address current problems with patient safety, complex health issues, and rising healthcare costs. In fact, health professions have now incorporated IPE and communication into the nursing curriculum and practice (Interprofessional Education Collaborative Expert Panel 2011; American Association of Colleges of Nursing 2008). Several studies documenting the importance of IPE for medical and nursing students identified successful outcomes including improved communication skills, increased knowledge of role, and greater self-efficacy (Norgaard et al. 2013; Tofil et al. 2014). If educating professionals together leads to such success, starting this process before students reach professional schools could lead to even greater improvements.

Professionals in biology tend to agree with the importance of collaborating with those with different areas of expertise and training. There is need to develop mechanisms that nurture collaboration and a mechanism to enhance collaboration at all educational levels, from undergraduates to senior faculty, may be interdisciplinary group projects (Miller and Alban 2012). Additionally, Fantini et al. (2011) state, “We strive to educate where the needs, opportunities, and jobs are and will be in the future. The bridge between engineering, biology, and medicine is a growing link, and there is no sign that this interface will slow.” Many biology undergraduates have plans to enter professional schools. Interprofessional education has been shown to positively change medical students’ attitude towards working in teams (Lyons 2012). Additionally, collaborative education between engineering and medical students has shown to result in innovation for community benefit through assistive device creation (McClelland and Kleinke 2013) and addressing real-world clinical problems (Spoelstra et al. 2014). We propose that these benefits might be achieved at the undergraduate level.

#### Effect of MakerSpaces on student learning outcomes

Space has been theorized to be a “change agent” within higher education since Oblinger (2006) summarized the physical and psychological theories guiding the importance of space on student learning. Oblinger (2006) further surmises that both the cognitive theory (the active construction of knowledge) and social constructivist theory, the idea that the social environment influences learning, support a learning environment allowing for decentralized interaction among peers. From a business perspective, fostering innovation requires removing traditional workplace boundaries and restrictions (and sometimes isolating the team from everyone else), and embracing the qualities (playfulness, openness, energetic, risk taking, etc.) of smart creatives (Berger and Brem 2016). Thus, we leveraged the MakerSpace as an instructional strategy (Fig. 1).

Despite widespread buy-in of these assertions, the science on space and student learning outcomes remains relatively sparse. One notable exception is the quasi-experimental research on two otherwise identical classes held in different spaces, which found that students in the classroom with a flexible design, work tables, and integrated technology had better student learning outcomes than those in the traditional classrooms with desks and an instructor lecture podium at the front of the classroom (Brooks 2011). A follow-up study from the same program of research also found that students in active learning environments outperform peers in traditional classroom environments (Cotner et al. 2013). Others have noted trends supporting better student learning outcomes in active learning classroom spaces, though results were not statistically significant (Muthyala and Wei 2012).

A 2016 literature review on the topic concluded that the research on space and student learning outcomes in higher education is “dispersed and fragmented” (Ellis and Goodyear 2016). Moreover, despite broad interest in educating higher education students in MakerSpaces, there is space research on student learning outcomes in higher education within these spaces. However, a recent study of Spanish engineering undergraduate students enrolled in a MakerSpace workshop used the Abreaction Test of Creativity (de la Torre (1991) to show gains in creativity (Saorín et al. 2017). This is a promising result and research on the learning outcomes beyond creativity and single discipline is needed.

### Significance of this study

Literature exists supporting the benefits and use of IPE education (Cuff 2013), yet a majority of this development is being done at the professional or graduate levels rather than in undergraduate settings, and certainly not early in their academic career. In our course, students did not work directly with a specific patient nor in a clinical setting; instead, the focus was on multidisciplinary collaboration in a technology-rich setting to solve pervasive health problems at the community level. Currently, no literature is available on the how well students achieve learning outcomes associated with multidisciplinary teamwork and emerging technologies nor how working in a technology-rich setting impacts attitudes and behaviors. Moreover, we found no existing literature on outcomes associated with students collaborating in the novel configuration of undergraduate student teams presented here, emphasizing the importance and relevance of our study.

## Methods

### Aim

The study aims to evaluate the impact of undergraduate multidisciplinary education offered in a MakerSpace on student attitudes towards and perceptions of skills required in their own as well as others occupations.

### Course design

The course was designed to educate a multidisciplinary group of students to meet the same learning outcomes (Table 1). The learning outcomes were established based on our experience and knowledge of professional competencies that teamwork is valued in the workplace but is rarely explicitly taught, and of many of our university’s students’ lack of exposure to emerging technology. The instructors facilitated students achieving these course objectives by creating a non-threatening learning environment, requiring weekly reflection, providing a relevant learning experience through a course project, and incorporating a variety of teaching and learning strategies (per Oandasan and Reeves 2005).

We provide a brief description of the course here, and a full description of the course is available in (Nagel, Lewis, and Ludwig 2017). Our students formed multidisciplinary teams according to their major (a distribution of majors on each team) and personal strengths (described below) with the aim to develop collaboration and problem solving capabilities. Gradable materials included weekly journals, tangible deliverables, an oral final exam, and presentation of a final prototype. The three professors (biology, engineering, and nursing) contributed by teaching, grading, and attending all class sessions, thereby providing a positive model for students.

The course was designed to teach students effective collaboration while utilizing cutting-edge technologies. Offered as a 3-credit elective, the class consisted of a weekly 3-h laboratory in which students play an active role in their learning, modeled after the Kolb learning cycle within a ME creative environment.

Each week students were assigned readings from one of four books (*Strengths Based Leadership (*Rath and Conchie 2008)*, Creative Confidence (*Kelley and Kelley 2013 *), Fabricated (*Lipson and Kurman 2013), and *The Immortal Life of Henrietta Lacks* (Skloot 2010) and responded to a reflective journal prompt before the class met. Throughout the semester, content learning topics included experts speaking about community health challenge of metabolic syndrome, principles of design thinking, how to use the equipment in the MakerSpace, intellectual property, fundamentals of a product pitch, and ethical reasoning. Teams were given at least 3 weeks of in-class work time in which the instructors acted as consultants. Teams provided feedback on their designs to each other and feedback to their teammates on their performance. The focus of the course was on the process, not the quality of the final product, so assessment was mainly formative except for progress benchmarks established at the beginning of the semester.

Prior to class, students took a StrengthsFinder® assessment (Rath 2007). This assessment comes with *Strengths based leadership (*Rath and Conchie (2008). This inventory helps identify 34 personal strengths or “talent themes” as they relate to personal behavior and performance. Students interviewed all other students in the class about the overlap of strengths, or lack thereof, and subsequently suggested the top two students from other majors that should be on their team in order build a team of diverse strengths. Ultimately, instructors formed finalized teams with every effort made to consider the recommendations of the students. Each student team contained representatives of each discipline. Students were then provided instruction and evidence on the importance of diversity in teams.

The course was offered in one of James Madison University’s first MakerSpaces. The space included a 3D printer, a laser cutter, a large format printer, virtual reality software, and basic craft supplies. Furniture in the space was flexible, and students could arrange and rearrange as their needs changed. There were multiple digital displays throughout the room around which students could gather in teams and work. Mobile dry erase boards were also available for student use. Students received instruction on how to use all the equipment in the room.

### Research design

#### Characteristics of participants

Eight students from engineering (a mix of undergraduate juniors and seniors), and seven students each from biology/pre-professional health (a majority of sophomores) and pre-nursing chronic illness minor students (all sophomores) were enrolled in the course (total *n* = 22) (Table 3). All students took the course as an elective. The biology/health sciences and engineering instructors recruited students directly based on prior interactions with the students. The pre-nursing students from the chronic illness minor were able to register for the class on a first-come first-served basis. Following institutional review board approval [IRB Protocol Number 16-0060], we recruited 22 student members of the course to participate in the research study. The participant sex breakdown reflected the institution’s ratio of 59% female; as age, gender, and ethnicity were not targeted in our research design, the transcripts reflect this de-identification.

**Table 3:** Number of participants by major and grade level

Pre-profession	Grade level			Total (*n*)	Percentage of group total (%)
	Sophomore (2nd year)	Junior (3rd year)	Senior (4th year)		
Engineering	0	5	3	8	36.4
Pre-nursing	7	0	0	7	31.8
Biology/health designate	6	1	0	7	31.8
Total	13	6	3	22	100.0

### Data collection

Data were collected as part of an exit interview at course end. We asked each student during individual interviews, “What did you value about what you learned in the class?” We prompted students during the ensuing discussion, audio recorded and transcribed the recordings, and removed all identifiers.

After all course grades were submitted, the transcripts were analyzed using qualitative content analysis (Graneheim and Lundman 2004) to identify themes in the student reflections. This method requires the researcher to reduce participants’ comments to their smallest meaningful unit, code these units, identify categories for these codes, and then finally distinguish themes from the categories. The three researchers used an iterative process until agreement was reached at each step to strengthen the trustworthiness of the findings, though no inter-rater reliability statistics were calculated, and counts maintained of codes, categories, and themes to enhance understanding of the reports of the qualitative data, per Sandelowski (2001). We used the comments from the end of the semester peer evaluations (Ohland et al. 2012) to further elucidate some themes, especially those around teamwork. These quotes are designated as “[iX]” in the results (Additional file 1).

### Design and analysis

We used qualitative content analysis to look for themes in students’ attitudes and learning in this multidisciplinary education course. As researchers, we attempted to avoid all biases when conducting our evaluation of data. Thus, we did not specifically look for the described objectives and domains (Table 1) during our research process. As discussed above, when we evaluated our student outcomes the overlap between the objectives and five domains of the Interprofessional Collaborative Practice Competencies (IPEC) became evident.

## Results

The narratives resulted in over 250 unique and coded meaningful units in which six themes emerged from over 50 categories. These categories included *learning to allow time for team work* and *gained confidence in professional knowledge*. Six themes were observed. Of these, two were emergent, with the remaining four aligning directly with course objectives and IPEC domains. The themes are discussed below in order of the volume of supportive evidence. The designators after the quotes signify an individual quote [iX] from the transcripts or a quote from the catme.org peer evaluation [cX].

### Learned teamwork capabilities and valued working in a multidisciplinary team

The two most supported themes, “learned teamwork capabilities” and the more specific “valued working in a multidisciplinary team” are related, though separate important foci exist. These themes align directly with the course objectives (Table 1). For instance, the theme of “learned teamwork capabilities” contained general capabilities required of participants to work well in a team, such as being purposeful to understand others’ perspectives, whereas the theme “valued working in a multidisciplinary team” illustrates that participants noticed the difference required to work with other disciplines, and the skills they consequently developed to make that happen. Both themes contained capabilities that went beyond knowledge to include skills, attitudes, and behaviors, as noted below. Furthermore, participants gained knowledge of their own strengths and weaknesses in relation to the team; they learned how to interact with others, and that teamwork takes time. They developed new beneficial practices and attitudes, such as patience. And perhaps most importantly, students came to appreciate the role of the team and realized that teamwork was necessary to the success of their eventual goals. The narratives below illustrate students learning general teamwork capabilities:

I believe that the Strengths Based Leadership assessment properly assigned our group. Each of us had individual strengths and talents that complemented each other. I have learned so much from my team members and though their contributions varied from time to time, I believe they all deserve perfect scores. This class also introduced me to new friendships. It was one of the few classes I have taken at JMU that I can say I genuinely enjoyed being a part of. It excites me to see how innovative my peers are and how JMU is supporting this creativity. [c1].

I learned that I can be really distracting in groups and that needs to be controlled, but it can also be. . . like, distraction in itself is not inherently negative. Sometimes you need a distraction. So if tensions were getting high, they didn’t in our group, but if things get to a fever pitch I can break away from that. And I think that adds something useful. The strengths-based leadership is helpful in that you should work on your strengths but I think it’s also possible to look at your weaknesses and frame them as strengths in the right light [i12].

I wanted to make this cool meat grinder that I thought was awesome. So I guess one of the main things I learned throughout this course mostly in the group context is like… myself I just wanna make the idea, do it myself and to hell with your ideas, right? But I realized after trying to convince them and they just weren’t having it, I realized they have a good idea. I was just too focused on my own idea to really give it a shot at first. And so, I don’t know, I learned to accept that maybe I don’t have to come up with all the ideas. I don’t have to dominate the group like I did earlier in the semester when I wouldn’t really let them talk per se. [i2]

In addition to working as a team, students understood that working in a multidisciplinary team was unique, and they specifically appreciated that dynamic. Students valued knowing what the other professions do, finding it humbling to recognize the expertise in their peers, and they learned to overcome practical barriers such as jargon differences. Likewise, they acknowledged that working specifically with a group from other professions led to a richer process, and students maturely valued that. Learning concepts related to interprofessional work are illustrated in the following participant comments:

It was difficult at first realizing that not everybody knows my lingo and I have to ask a lot people in a different way all the time, and I definitely value that I now feel comfortable being like, Hey, I don’t get that, can you tone it down a bit for me and really break it down so I can grasp what you’re saying and we can move forward? [i9]

Okay. I think what I learned a lot in the class is how to deal with the whole inter-professional aspect because like when you’re in the workforce, ideally or not ideally, you’re going to be surrounded by different people with different backgrounds and that’s what this class was so it kind of taught me, and I feel like everyone else, to get a different perspective or to understand someone else’s perspective on what they’re trying to pitch or voice to you. [i14]

My team was great, we worked well, all members participated in all activities and expressed enthusiasm about the project. I cannot express how awesome it’s been to work with non-engineering majors, I saw great attitude from the Biology and Nursing students! [c4].

### Valued growth from challenging problem solving

Students valued the growth that came from being challenged in the course; this theme aligned with the course objectives (Table 1). Though they called the growth uncomfortable, participants recognized that the MakerSpace and interprofessional aspects allowed for more robust problem solving. The students noted the challenge of having the course instructed differently than many they had taken prior; they commended the student-centered approach to teaching which allowed students more autonomy to direct decision-making. They appreciated the self-directed nature of the course. One participant describes how the student-centered learning was integrated into the creative aspect of the course. They also enjoyed learning about the health challenge(s) of metabolic syndrome. Students sensed an urgency to their work and the access to new technology pushed them to grow and problem solve. In fact, some specifically mentioned the 3D printer as a useful technology:

I didn’t realize we were going to be using 3D printers and laser cutters and all this cool technology, so I think I really kind of learned to expand past my typical lecture science brain to a different kind of field [i10].

…but it was also just cool to learn about the actual innovation process and how to get from just an idea that I wrote down on a piece of paper to actually up to something that was working and could almost be put into an actual product. [i15]

[Names] were one of the best teams I’ve ever worked with at JMU! Even when we had conflicting ideas, everyone was eager to listen to everyone else and work together to create something we ended up being really proud of. My favorite thing about working with them was that instead of splitting up all the work and doing it individually, we all spent a lot of time working as a group and meeting outside of class. So, we all ended up being on the same page. Creating our project was tough because we were going through new ideas every week for a while, but everyone in the group kept going and giving suggestions on how to make it better. And, when we were on a good track, everyone was equally excited! We had a good dynamic within our team, and I think our differing personalities worked well together. [c2]

### Saw the course in the context of their past and future work

An emergent theme described how students saw the course in the context of their past and future work. This was an affirmation to us as facilitators/educators of the importance of the class to their learning. Students mentioned that they enjoyed the course and that they will be able to apply what they learned to future work. Additionally, they linked their class activities to other non-academic experiences. For example, participants reported being excited to tell their families and peers about the class:

You guys gave us useful information and resources, and kind of let us utilize and at our own will. And that was really cool, because I’ve never had anything like that, especially being a health science major. It’s like preparing you for further schooling, so they don’t think that you need to have a lot of hands-on things. We don’t get a lot of that within our major. So that was something that I really valued a lot. [i7]

And I [talk] about this class all the time, to like my parents and friends and stuff, but I really hope that continues to keep the things I’ve learned, into my other future bio classes or even just non bio classes because it truly, creativity can be fostered, and that’s a big thing I learned. [i13]

### Learned about their own profession

The students reported that they acquired insight into their own profession, a theme that aligned with the course objectives (Table 1). These undergraduates also learned the limitations of their profession and at the same time were able to gain confidence in their professional knowledge. Moreover, they learned to communicate their role and their knowledge to other members of their team. Markedly, students came to see that they are more than “just” their chosen profession:

And I’ve learned about professionalism outside of me but I’ve also learned about internal professionalism within myself. Like, “What does it mean for me to be all these different things at once?” and “How do I access that?” I don’t have to be. . . I mean, yes, I’m a biology major. That’s what I’ll graduate with, but I’m not just a bio major. [i12]

### Learned to be creative

Students also gained knowledge of how to be creative (an emergent theme); many were previously unaware of their capabilities. In this experimental course they realized that creativity can be a learned skill. Participants valued the innovative thinking and increased the skill of ingenuity while revering the structure the course provided for learning creativity and felt that benchmarking was useful to provide feedback of their creative work.

I think it really helped me to be more creative and just have more confidence in my abilities and my ideas [i6].

Another participant describes the creative growth that occurred:

I really learned to not doubt myself so much when it comes to creativity. I’ve always never really thought of myself as creative and I guess being a science major people don’t think of you as creative when you tell them that. But I really got to tap into the part of me that is more creative and artsy and crafty and looks at things in a different perspective, and I talked about that in my last journal [i13].

## Discussion

This initial investigation of student learning gains in the areas of multidisciplinary teamwork in a MakerSpace suggests that the innovative configuration of majors and learning environment was effective. Students reported a deepened understanding of their own discipline and an increased appreciation for the future career paths of their teammates, as exemplified above in quote i12. The emanating themes of students valuing teamwork—especially multidisciplinary teamwork, exploring exciting new territory in their own professions, and learning to be creative both corroborate prior findings and add to the knowledge of IPE for our increasingly collaborative world. We interpret our results in the context the IPEC domains and of our learning objectives.

The interprofessional teamwork domain focuses on building relationships to provide patient care. This domain aligns with our course objective of “Use collaboration skills to work together with a group of diverse pre-professionals”. Although our students did not participate in direct patient care, they demonstrated capabilities needed to work as an interprofessional team. For instance, students integrated the knowledge of other professions into their decision-making process. The quote [c2] makes this case quite clearly; students worked in tandem to solve problems rather than use the divide and conquer method.

The IPEC domain of “roles/responsibilities” states that professionals need to develop a sense of their own profession (what skills and knowledge are unique to the profession) along with valuing interprofessional work. Our students were able to articulate steps of professional identity development, such as understanding professional boundaries, despite the fact that in some cases they had not even begun formal professional training. In addition, many students were being educated on their role while also developing interprofessional competencies. This observation is important, because others have suggested that it is critical to first develop a professional identity before developing interprofessional competencies (Khalili et al. 2013). In comparison, our findings suggest that this process may be nonlinear and that the two roles can be learned simultaneously, reiterating our study’s innovative approach of introducing undergraduate students to the world of professional collaboration.

The interprofessional communication domain includes communication with team members that do not share one’s specific occupation. This domain is characterized by recognizing one’s expertise and expressing opinions, along with choosing effective communication techniques. This domain aligns with our course objective of “Produce and communicate a tangible product using MakerSpace technology that has the potential to improve a community health challenge”. Our findings support that undergraduate pre-professional students can learn communication skills, such as overcoming jargon barriers, needed to work in a diverse team. An interesting outcome shown in quote [i9] is that this student articulated being better able to ask clarifying questions as a function of working in interprofessional teams.

One IPEC domain that the instructors expected the student responses to support, but was missing in the student narratives, was the values/ethics domain, which discusses ethical decision-making in the context of interprofessional practice and suggests that ethical decisions often require team input. This domain includes empathy, an emphasis that the patient is the center of the team and of all decisions made (Interprofessional Education Collaborative 2016). In the current team setting, the “client” for whom the students designed their solution would take the place of the patient. Curiously, our students failed to articulate the importance of ethical decisions in the context of the interprofessional team despite receiving instruction on, and content in, diversity and ethical decision making. For example, students read, discussed, and reflected on the application of the book *The Immortal Life of Henrietta Lacks* (Skloot 2010) in regards to other course content. Students struggled to see the connection between the theme of human vulnerability to technology in the book and in the novel technologies we used in the course. Instead, students tended to be focused on the practical details of the technology, such as how to use the large format printer. This result suggests that within the context of developing new, complex, technical skills, e.g., working with a 3D printer, it may be difficult for undergraduate students to transition to and value higher-order thinking skills such as ethical decision-making. Therefore, we recommend substantial scaffolding in course assignments to help students develop ethical reasoning in the context of interprofessional practice.

Although *students* failed to identify gains in ethical reasoning, the *instructors* perceived at least one area of ethical reasoning in which students made gains was in the development of empathy. We noticed that students improved in their ability to design for others as the course progressed. Students learned and used an empathy-based design process as described in *Creative Confidence* (Kelley and Kelley 2013) which provides principles and strategies for unlocking one’s creative potential, as well as innovative ways to approach and solve problems. Empathy-based design is an iterative process used to create a solution that has value because the designers take time to understand the people not only as someone with specific health needs but who they are as a person, resulting in a comprehensive process that caters to the individual’s needs. The topics of novel technology and multidisciplinary practice require ethical reasoning skills to be well-implemented. Empathy is only one component of ethical reasoning, and it is unlikely that understanding of empathy-based design is sufficient for ethical reasoning. Thus, in addition to emphasizing the importance of empathy when teams are formulating their design, explicit instruction was provided on ethical reasoning that included considering elements such as outcomes and autonomy (Madison Collaborative 2013). Though students described their learning specific to empathetic design when they talked about the space and problem solving, they failed to make the connection between empathetic design and ethical reasoning. We believe that empathy-based design has the potential to play an important role in developing ethical reasoning for undergraduate interprofessional teams and suggest these learning goals be as explicit as possible to students so that they can specifically identify the value of, and intentionally hone, this skill during the course, while recognizing the need for future focus on ethics as they work in subsequent interprofessional teams.

### Student learning gains

There is empirical evidence that the space in which students learn may also have an impact on learning outcomes; our research supports Mellor et al. (2013) findings that for IPE, the educational environment is important to student learning, and specifically that it is crucial for students to have a space in which to try, and fail, and have the ability to work on realistic problems. In particular, MakerSpaces may facilitate student creativity and may help explain the emergent theme of students learning the skill of creativity. Students identified the MakerSpace as inspiring, relevant, and motivating (see quotes [i10], [i15], [c2]), however the students did not link the space directly to facilitating multidisciplinary collaboration. Our results provide initial evidence to the philosophical argument that MakerSpaces are places in which students learn creative problem solving, leverage their strengths, and take ownership of their learning (Kurti et al. 2014) but further research is required linking students perceptions of the space directly to their learning.

Sheridan et al. (2014) state, “Makerspaces seem to break down disciplinary boundaries in ways that facilitate process- and product-oriented practices…” This was a technological introductory experience for our students, as the MakerSpace in which the course was offered was a new facility, and even if the students had experience with 3D printing, they had not used these particular brands and machines. The acts of learning to use the equipment, trouble-shooting errors together, and celebrating successes can all lead to the space having a strong influence on collaborative problem solving. And while university libraries across the USA are installing MakerSpaces, these iterations fail to promote collaborative learning because they are typically for use by individuals (Gierdowski and Reis 2015). Additionally, library MakerSpaces do not explicitly promote the reflective portion of the Kolb learning cycle (Fig. 1). Contrasted with the library MakerSpaces, there are examples of MakerSpaces dedicated to single disciplines. A study of a MakerSpace in engineering showed a positive impact on professional growth, a slightly less but still positive impact on personal growth, and a large proportion of their growth in the area of teamwork (Lagoudas et al. 2016) for students. Our results are in line with those findings and broaden them with the additional component of multidisciplinary learning.

We can speculate additional reasons for the student perceptions of their learning gains in the course. One reason for these advances might be the choice of course texts the students read; the texts were intentionally chosen to guide students through an exploration of teamwork and creativity, the ethics of their potential interventions, and the culture of 3D printing. Interestingly, however, students did not often reference the books directly in their interview responses. A second explanation could be that the course strongly aligns with the emergent themes of the “smart creative” (Berger and Brem 2016) including seeing failure as an opportunity, recognizing the need for technical knowledge and creativity, and that problem solving involves the generation of multiple ideas.

A third explanation for these outcomes might be attributed to areas that students did not identify as impacting their classroom experience. For instance, the instructors noted, anecdotally, that many teams began to meet socially for non-class related purposes. The students did not report these interactions as being important in their exit interview, thus it did not emerge as a theme. However, these informal interactions may have helped them deepen their respect, communication, and understanding for the distributions of various capabilities and lack thereof held in their team. These informal interactions, although admittedly difficult to study directly, may play an important part in the success of the team (Lawson et al. 2009).

The potential impact of the instructors on the outcomes of this research, however, is less speculative. An evaluation of a National Science Foundation (NSF) Research Experience for Undergraduates at University of California—Irvine that focused on interdisciplinary teams in health-related areas found that the attitudes of the mentors predicted attitudes of participants towards interdisciplinary work (Misra et al. 2009). The team of instructors for this research has considerable overlap in their strengths (including strategic thinking and relationship building), as well as remarkable differences in areas such as executing and communicating. Each one highly values interprofessional work, and have all been through extensive professional development practices. Moreover, professional development has shown to have had a profound impact on the standard of “Facilitating Learning and Creativity” in a study of 32 teachers using the International Society for Technology Education National Educational Technology Standards (NETS•T) and Performance Indicators for Teachers (Fiala et al. 2015). Thus, as instructors in this study, we may have been especially well-positioned to facilitate student learning and promote the value of working in multidisciplinary teams.

### Limitations

Limitations of our study include our data set being collected orally only by instructors. Though students were assured this would not be graded and all identifiers removed, participants may have been hesitant to share feedback in front of the group, particularly if their insights could be construed as negative or involving team members directly. An external interviewer for the exit interview would eliminate this potential bias. Additionally, a written component should be considered in the future to gather additional unbiased data.

We note that there is a relatively small sample size in this study and we did not collect data with regard to ethnicity, race, or gender identification. A next step would be to increase the sample size and analyze the results relative to these parameters within our own institution. Additionally, we suggest including multiple institutions with various Carnegie classifications and replicating the study through multiple cohorts to broaden the applicability of the results. Longitudinal studies including students’ perceptions of how the class influenced their current thinking and employment would contribute to the understanding of the importance of these types of experiences for student outcomes.

### Next steps: qualitative to quantitative data

We intended this research to be qualitative in nature because we were not sure what to expect from students in this course experience. We have already used the emergent themes from this qualitative assessment of student learning gains to select quantitative assessments of the noted improvements for the next offered iteration of the course, however, those results are not available yet. Quantitative surveys have been offered to undergraduate students in medical and engineering disciplines in order to uncover their attitudes and preferences regarding working and learning across disciplines (Spoelstra et al. 2014). Unfortunately, however, there is no data on the change in those preferences after working in an interprofessional team. The next iteration of the course incorporates a before and after survey to address this issue. Additionally, there are studies that evaluate the efficacy of interprofessional programs, but do not directly assess students’ achievement of learning outcomes (Simmons et al. 2016). This is a more challenging quantitative assessment because it shifts from student reporting to instructor assessment of performance. Future iterations of the course could use the AAC&U value rubrics to assess performance. Ideally, longitudinal studies will eventually be done to determine if the student gains in the course last through their professional training and future work, including specific measurement on students’ capacity to acquire emerging content knowledge to stay on the cutting edge of their rapidly changing fields.

## Conclusions

In a MakerSpace, we simultaneously guided and watched our students grow in their confidence and mastery of capabilities such as teamwork and communication, which are crucial to collaboration and in demand by employers. Students valued the multidisciplinary nature of the work, their own “professional” and personal development, and the technologies available to them. These observations align with the course objectives that also aligned with the IPEC domains, and provide evidence that the overlap between multidisciplinary teams, authentic problems is a valuable pedagogical space for training current undergraduate students for future professional work. The MakerSpace was inspirational to students however, students did not make a direct connection between the space and their learning. More explicit research is required to determine the effect of the MakerSpace on student learning gains; however, the outlook is promising. Additional research is also needed to establish evidence-based quantitative measures of proficiencies in each objective. We assert that specifically focusing on personal strengths and exposing students to tough questions are equally important, as is emphasizing ethics throughout the creative course process. Finally, novel configuration of teams in technology-rich environments is a promising area for future work.

## Additional file

Additional file 1: Themes and categories listed in descending order based on volume of supportive evidence. (DOCX 14 kb)

## Abbreviations

AAVMC: Association of American Veterinary Medical Colleges
ACAPT: American Council of Academic Physical Therapy
APA: American Psychological Association
CSWE: Council on Social Work Education
IPE: Interprofessional Education
IPEC: IPE Collaborative
JMU: James Madison University
NSF: National Science Foundation
STEM: Science, technology, engineering, and mathematics
