# Supplementary material for: Student learning outcomes from a pilot medical innovations course with nursing, engineering, and biology undergraduate students
Source: Int J STEM Educ. 2017 Nov 27;4(1):33. doi: 10.1186/s40594-017-0095-y (PMC6310392; doi:10.1186/s40594-017-0095-y)
Supplement: Supplementary file 1 — Themes and categories listed in descending order based on volume of supportive evidence. (DOCX 14 kb) [file 40594_2017_95_MOESM1_ESM.docx]

**Additional file 1**

**Themes and Categories listed in descending order based on volume of supportive evidence**

**Theme**

Category

**Learned teamwork skills**

Learned to effectively work as a team

Learned how to work with differing perspectives

Learned about personal strengths and weaknesses

Importance of teamwork to success of projects

Learned specific skills such as patience that made teamwork easier

Appreciated teamwork

Learned how to interact with others

A team experience is unique

Learned to work with people with different skills

Learned to allow time for teamwork

Personal weaknesses can also be strengths

**Valued working in an interprofessional work**

Learned the knowledge and skills of other professions

Valued interprofessional nature of the work

Valued having multiple instructors

Learned from other professionals

The interprofessional team led to a richer process

Learned to overcome jargon barriers between different professions

Humbling to recognize the expertise of others

Comfortable working in interprofessional teams before the class

**Valued growth that came from challenges of problem solving**

Uncomfortable growth

Valued MakerSpace

Learned about the health challenge

Learned about technology

Valued having a deliverable product

Learned empathy for design

3D printing

Learned new skills

Saw limitations of maker-space

Limited technical communication

Course self-directed

Class different than expected

Student centered approach

Valued being off campus for class

Sense of urgency to work

Information was valuable

**Saw the course in context of past and the future work**

Linked class to other experiences

Will be able to apply class to future work

Enjoyed the class

Learning was limited

Strengthened prior knowledge

Used prior knowledge to meet the challenge

Hoped to continue learning more about course content

Learned to learn

**Learned about own profession**

Learned limitations of own profession

Learned about own profession in general

Gained confidence in professional knowledge

Learned that I am more than just my chosen profession

Learned to communicate professions role and knowledge

**Learned to be creative**

Increased creativity skills

Creativity can be learned

Valued innovative thinking

Valued the complexity of the work

Previously unaware about creative abilities

Appreciated the structure to the creative work

Benchmarking was useful
